# Diagnostic tests and treatment procedures performed prior to cardiovascular death in individuals with severe mental illness

**DOI:** 10.1111/acps.13157

**Published:** 2020-02-29

**Authors:** I. H. Heiberg, R. Nesvåg, L. Balteskard, J. G. Bramness, C. M. Hultman, Ø. Næss, T. Reichborn‐Kjennerud, E. Ystrom, B. K. Jacobsen, A. Høye

**Affiliations:** ^1^ Center for Clinical Documentation and Evaluation (SKDE) Tromsø Norway; ^2^ Norwegian Medical Association Oslo Norway; ^3^ Department of Clinical Medicine UiT – The Arctic University of Norway Tromsø Norway; ^4^ Norwegian National Advisory Unit on Concurrent Substance Abuse and Mental Health Disorders Innlandet Hospital Trust Hamar Norway; ^5^ Department of Medical Epidemiology and Biostatistics Karolinska Institutet Stockholm Sweden; ^6^ Icahn School of Medicine Mt Sinai Hospital New York NY USA; ^7^ Institute of Clinical Medicine University of Oslo Oslo Norway; ^8^ Institute of Health and Society University of Oslo Oslo Norway; ^9^ Department of Mental Disorders Norwegian Institute of Public Health Oslo Norway; ^10^ Department of Psychology PROMENTA Research Center University of Oslo Oslo Norway; ^11^ PharmacoEpidemiology and Drug Safety Research Group School of Pharmacy University of Oslo Oslo Norway; ^12^ Department of Community Medicine UiT – The Arctic University of Norway Tromsø Norway; ^13^ Department of Community Medicine Centre for Sami Health Research UiT – The Arctic University of Norway Tromsø Norway; ^14^ Division of Mental Health and Substance Abuse University Hospital of North Norway Tromsø Norway

**Keywords:** schizophrenia, bipolar disorder, cardiovascular diseases, death, delivery of health care/standards

## Abstract

**Objective:**

To examine whether severe mental illnesses (i.e., schizophrenia or bipolar disorder) affected diagnostic testing and treatment for cardiovascular diseases in primary and specialized health care.

**Methods:**

We performed a nationwide study of 72 385 individuals who died from cardiovascular disease, of whom 1487 had been diagnosed with severe mental illnesses. Log‐binomial regression analysis was applied to study the impact of severe mental illnesses on the uptake of diagnostic tests (e.g., 24‐h blood pressure, glucose/HbA1c measurements, electrocardiography, echocardiography, coronary angiography, and ultrasound of peripheral vessels) and invasive cardiovascular treatments (i.e., revascularization, arrhythmia treatment, and vascular surgery).

**Results:**

Patients with and without severe mental illnesses had similar prevalences of cardiovascular diagnostic tests performed in primary care, but patients with schizophrenia had lower prevalences of specialized cardiovascular examinations (prevalence ratio (PR) 0.78; 95% CI 0.73–0.85). Subjects with severe mental illnesses had lower prevalences of invasive cardiovascular treatments (schizophrenia, PR 0.58; 95% CI 0.49–0.70, bipolar disorder, PR 0.78; 95% CI 0.66–0.92). The prevalence of invasive cardiovascular treatments was similar in patients with and without severe mental illnesses when cardiovascular disease was diagnosed before death.

**Conclusion:**

Better access to specialized cardiovascular examinations is important to ensure equal cardiovascular treatments among individuals with severe mental illnesses.

**Significant Outcomes**

- Patients with schizophrenia had a lower prevalence of specialized cardiovascular examinations. Patients with schizophrenia or bipolar disorder had lower prevalences of invasive cardiovascular treatment prior to cardiovascular death, implying a need for better efforts to prevent, detect, and treat cardiovascular disease in these individuals.
- The prevalence of invasive cardiovascular treatment did not differ between individuals with or without severe mental illnesses when cardiovascular disease was diagnosed prior to death.
- Underuse of specialized somatic health care is probably an important explanation for the increased cardiovascular mortality observed in patients with severe mental illnesses.

**Limitations**

- The diagnostic quality has not been established for all causes of death and all disease categories.
- We lacked data on physicians' assessments of risk factors along the clinical pathway and data on referrals and patients' possible non‐consent to clinical examinations and treatment.
- The level of screening/monitoring of cardiovascular risk factors may be underestimated, as we lacked precise information on the indication and content of some common diagnostic tests included in broad batteries of biomarker assessments.

## Introduction

Equal access to health care for equal medical needs is a core value in countries with universal healthcare systems. Individuals with schizophrenia or bipolar disorder (subsequently referred to as severe mental illnesses, SMI) have a higher prevalence of risk factors for cardiovascular disease (CVD) 1, a higher cardiovascular morbidity 2, and an increasing mortality because of CVD compared with the general population 3. Nevertheless, meta‐analyses report inferior metabolic monitoring in individuals with SMI 4 and a lower likelihood of hypertension treatment 5, cardiovascular prescriptions 6, and invasive coronary procedures 7. A lower likelihood of cardiovascular risk factor monitoring 8, cardiovascular prescriptions 9, 10, 11, 12, 13, or acute treatment of CVD 14, 15, 16 has also been reported in comparative studies from countries with universal health care. Some recent studies, however, report similar access to treatment following acute myocardial infarction (MI) in patients with and without SMI 17, 18, and a higher likelihood of statin prescriptions 19 and revascularization 20 in younger individuals with SMI compared to controls.

A recent meta‐analysis noted that the stage along the pathway of care at which individuals with SMI lose access to cardiovascular examination and treatment is unclear 21. Earlier studies have reported lower 8, 13, 22, 23, similar 13, 22, 23, 24, and higher 13, 24, 25 use of CVD‐related diagnostic tests in primary care settings among individuals with SMI. With the exception of post‐MI treatments, few studies have assessed the number of examinations and treatments for CVD performed in specialized healthcare settings for individuals with SMI. Additionally, few studies have reported the rates at which CVD‐related diagnostic tests and cardiovascular treatments are administered in the more severe phase of CVD (i.e., in the period prior to cardiovascular death). To the best of our knowledge, no prior nationwide studies of the prevalence of diagnostic tests and invasive treatment of CVD in individuals with schizophrenia or bipolar disorder have been performed across healthcare sectors.

### Aims of the study

In this study, we examined whether a diagnosis of schizophrenia or bipolar disorder was associated with a lower prevalence of diagnostic testing and treatment for cardiovascular disease prior to cardiovascular death. A secondary aim was to investigate whether any disparity in healthcare use because of severe mental illnesses differed between primary and specialist healthcare settings.

## Materials and methods

### Study design and data sources

We conducted a national mortality follow‐back prevalence study that included all residents in Norway who died from CVD during 2011–2016. The dataset contained information on cardiovascular mortality and complete data on diagnoses and healthcare use in primary care and specialized in‐ and out‐patient settings. Mortality data were obtained from the Norwegian Cause of Death Registry (subsequently referred to as the Cause of Death Registry), diagnostic and health service use data were obtained from the Norwegian Patient Registry (subsequently referred to as the Patient Registry), and data for the for control and payment of health reimbursements in primary care were obtained from the Norwegian Directorate of Health system (the KUHR database, subsequently referred to as the reimbursement database).

The Cause of Death Registry provides almost complete (98%) information on causes of death based on death certificates coded by the physician who examined the deceased 26, supplemented by autopsy data in approximately 8% of the cases. The Patient Registry contains information about all specialized healthcare settings (i.e., government‐owned hospitals and out‐patient clinics, and private health clinics with governmental reimbursement) from 2008. Data from substance abuse treatment facilities were available from 2009. Because of technical problems when reporting data, approximately 15% of publicly funded contacts at private somatic health clinics were missing in the study period 27.

The reimbursement database contains information on all patient contacts with general practitioners (GPs) who receive governmental reimbursement, including visits at the doctor's office or the patient's home, telephone contacts, emergency room visits, and laboratory tests. The reimbursement database is approximately 100% complete, as GPs are mainly funded on a fee‐for‐service basis.

Diagnostic codes in the Cause of Death Registry and the Patient Registry follow the International Classification of Diseases and Related Health Problems, 10^th^ Revision (ICD‐10), while diagnostic codes for primary care contacts follow the International Classification of Primary Care, 2^nd^ edition (ICPC‐2). Accurate linkage across registries was obtained using an encrypted personal identification number included in all registries.

### Subject inclusion criteria and diagnostic and procedural categories

We identified all deceased residents in Norway who were aged 18 years or older with a cardiovascular disease (ICD‐10 codes I00‐I82) recorded as underlying cause of death in 2011–2016. We then examined the data for these subjects for at least three years prior to death in the Patient Registry and the reimbursement database. Individuals registered as having died abroad (*N* = 132) and individuals with no healthcare contact during the period (*N* = 66, none of whom had a diagnosis of schizophrenia or bipolar disorder registered on the death certificate) were excluded.

Patients were included in the SMI group if a diagnosis of schizophrenia (ICD‐10 code F20/ ICPC‐2 code P72) or bipolar disorder (ICD‐10 codes F30‐F31/ICPC‐2 code P73) was recorded in the Patient Registry or the reimbursement database between 2008 and 2016 or in the Cause of Death Registry.

Patients diagnosed with both conditions (*N* = 94) were included in the schizophrenia group only. A diagnosis of CVD was considered present if ICD‐10 codes I00‐I82 or G45, or corresponding ICPC‐2 codes (K70‐K71, K74‐K80, K82‐K84, K86‐K87, and K89‐K94) were recorded in the Patient Registry or the reimbursement database between January 1, 2008, and the time of death.

We used three composite measures of CVD‐related procedures as the main endpoints: (i) any CVD‐related diagnostic test in a primary care setting, (ii) any CVD‐related diagnostic examination in a specialized healthcare setting, and (iii) any invasive cardiovascular treatment procedure (see a description of the included procedures in Table 1). Information on pharmacological treatments was not available.

**Table 1:** Description of the included CVD‐related procedures

Outcome measure	Procedure
Diagnostic tests in primary care settings	Electrocardiography (ECG)
	24‐h blood pressure measurement
	Total cholesterol test
	Blood glucose test
	Glycated hemoglobin (HbA1c) test
Diagnostic examinations in specialized care settings	Ordinary or 24‐h blood pressure measurement
	Electrocardiography (ECG)
	Echocardiography
	Coronary angiography
	Right‐sided heart catheterization
	Invasive electrophysiological examination of the heart
	Ultrasound examination of blood vessels†
	Doppler pressure measurements
Invasive cardiovascular treatment	
Coronary revascularization	Percutaneous coronary intervention (PCI)
	Coronary artery bypass graft (CABG)
Arrhythmia treatment	Permanent transvenous cardiac pacemaker implant
	Cardioverter‐defibrillator implant
	Electroconversion of cardiac arrhythmia
	Transvenous ablation
Heart valve replacement	
Vascular surgery	Carotid surgery
	Aneurysm surgery
	Peripheral vessels surgery

^†^ Includes only procedures conducted in medical departments. Information on procedures conducted in radiology departments was not available.

### Analysis and statistical methods

We applied a log‐binomial regression analysis to study the association between the presence of SMI and provision of diagnostic tests or treatment for CVD in primary or specialized somatic healthcare settings 28. The log‐binomial model for the use of diagnostic tests in primary care settings did not converge. We therefore used a Poisson regression analysis with a robust error variance for estimation in this particular study 29.

Sample characteristics were presented as crude numbers, with significant associations marked in bold after adjusting for age and sex. The log‐binomial models were adjusted for sex and age at death (age groups 18–59, 60–69, 70–79, 80–89, and 90 years and above), and presented as prevalence ratios (PRs) with 95% confidence intervals (CI). Models that also included substance abuse (see the definitions in Table S1) and somatic comorbidities according to the Charlson comorbidity index 30, 31 did not significantly change the estimates or improve the goodness of fit of the model, and thus are not shown.

We conducted analyses of all subjects and analyses stratified by sex for the most common CVD‐related diagnostic tests performed in primary care settings (i.e., electrocardiography (ECG), 24‐h blood pressure measurement, blood glucose test, and glycated hemoglobin (HbA1c) test) and the most common CVD‐related diagnostic examinations performed in specialized care settings (i.e., echocardiography, coronary angiography, and ultrasound of peripheral vessels). Finally, we conducted subgroup analyses of the receipt of revascularization (in all subjects and among patients diagnosed with ischemic heart disease), receipt of invasive arrhythmia therapy (in all patients and among patients diagnosed with arrhythmia), and receipt of vascular surgery (in all patients and among patients diagnosed with peripheral vascular disease or carotid artery stenosis (see the diagnostic codes in Table S1)).

We conducted sensitivity analyses to examine the effects of (i) excluding patients diagnosed with dementia, (ii) excluding patients aged ≥80 years, (iii) excluding patients with the ambiguous affective disorder diagnosis in ICPC‐2 (code P75) from the bipolar group, (iv) including patients where CVD was listed as a contributing factor but not the main cause of death, (v) adjusting for person‐years of observation, (vi) restricting analyses of invasive CVD treatments to patients who survived their first CVD contact in the study period, and (vii) excluding CVD diagnoses registered only during the last month of life from the subgroup analyses of revascularization, invasive arrhythmia therapy, and vascular surgery.

Data management and analyses were conducted using SAS statistical software, version 9.4 (SAS Institute Inc., Cary, NC). A *P*‐value < 0.05 was considered statistically significant.

The legal basis and exemption from professional secrecy requirements for the use of individual health data was granted by the Regional Committee for Medical and Health Research Ethics (2014/72/REK nord).

## Results

### Characteristics of the study population

We included 72 385 individuals aged 18 years and older who died from CVD in the period 2011–2016 and had visited primary or specialized somatic healthcare clinics at least once in the period from January 1, 2008, until death (Table 2). Of these individuals, 814 (1.1%) patients were diagnosed with schizophrenia and 673 (0.9%) were diagnosed with bipolar disorder. Approximately half (54%) of the patients with schizophrenia and 45% of patients with bipolar disorder had their SMI diagnosis recorded in a primary care setting only, while 13% of patients with schizophrenia and 28% of patients with bipolar disorder had their diagnosis recorded in a specialized healthcare clinic only.

**Table 2:** Description of the characteristics of individuals with schizophrenia, bipolar disorder, or no severe mental illnesses (SMI) who died because of cardiovascular disease (CVD) at ages 18 and older

	Schizophrenia	Bipolar disorder	No SMI
Demographic characteristics			
Persons, *n*	814	673	70,898
Men, *n* (%)	384** (47.2)**	291** (43.2)**	32,962 (46.5)
Age at death, mean (SD)	**76.0** (14.6)	**75.6** (13.4)	83.5 (11.3)
Aged 18–59 at death, *n* (%)	122** (15.0)**	83** (12.3)**	3,065 (4.3)
Aged 60–69 at death, *n* (%)	145** (17.8)**	129** (19.2)**	5,423 (7.6)
Aged 70–79 at death, *n* (%)	153** (18.8)**	157** (23.3)**	10,844 (15.3)
Aged 80–89 at death, *n* (%)	237** (29.1)**	201** (29.9)**	27,481 (38.8)
Aged 90 or older at death, *n* (%)	157** (19.3)**	103** (15.3)**	24,085 (34.0)
Causes of death, *n* (%)			
I20‐I25 Ischemic heart disease	327 (40.2)	252 (37.4)	25,806 (36.4)
I30‐I52 Other forms of heart disease	223 (27.4)	169 (25.1)	20,420 (28.8)
I60‐I69 Cerebrovascular disease	184 (22.6)	167 (24.8)	16,514 (23.3)
Other cardiovascular diseases	80** (9.8)**	85 (12.6)	8,158 (11.5)
Time from CVD diagnosis until death			
First CVD diagnosis at date of death, *n* (%)	139** (17.1)**	76 (11.3)	5,562 (7.8)
First CVD diagnosis ≤30 days before death, *n* (%)	186** (22.9)**	114 (16.9)	7957 (11.2)
Years from the first CVD diagnosis to death, median (25th‐75th percentiles)	**4.3** (2.4–6.0)	**4.2** (2.7–6.2)	4.8 (3.2–6.5)
Diagnoses, *n* (%)			
Cardiovascular disease	698** (85.7)**	610 (90.6)	66,351 (93.6)
Hypertension	390** (47.9)**	384 (57.1)	43,341 (61.1)
Cardiac arrhythmia	298** (36.6)**	262** (38.9)**	35,492 (50.1)
Congestive heart failure	333 (40.9)	257 (38.2)	34,186 (48.2)
Myocardial infarction	238** (29.2)**	183** (27.2)**	24,278 (34.2)
Cerebrovascular disease†	295 (36.2)	275 (40.9)	29,986 (42.3)
Valvular disease	114** (14.0)**	116** (17.2)**	16,367 (23.1)
Peripheral vascular disease	95** (11.7)**	101** (15.0)**	14,242 (20.1)
Pulmonary circulation disease	58 (7.1)	62 (9.2)	5,043 (7.1)
Hyperlipidemia	73** (9.0)**	84 (12.5)	7,525 (10.6)
Diabetes	186 (22.9)	156 (23.2)	14,726 (20.8)
Obesity	36 (4.4)	51** (7.6)**	1489 (2.1)
Chronic obstructive pulmonary disease	151 (18.6)	147** (21.8)**	11,147 (15.7)
Alcohol abuse	46 (5.7)	90** (13.4)**	2,283 (3.2)
Drug abuse	37** (4.5)**	64** (9.5)**	728 (1.0)
Modified CCI, mean (SD)	1.60 (2.0)	1.65 (2.1)	1.67 (2.2)
No CC groups, *n* (%)	341 (41.9)	250 (37.1)	28,232 (39.8)
≥2 CC groups, *n* (%)	219 (26.9)	165 (24.5)	18,010 (25.4)
Primary care usage per person‐year, median (25th‐75th percentiles)			
Contacts in primary care	**12.94** (6.46–22.87)	**15.79** (9.25–26.18)	11.44 (11.44–5.92)
No. of contacts according to type			
GP visits	**5.94** (2.60–11.95)	**9.25** (5.08–15.42)	7.00 (3.38–12.40)
Emergency room visits	**0.40** (0.17–0.91)	**0.50** (0.22–1.09)	0.35 (0.15–0.74)
GP telephone contacts	**4.57** (2.00–9.20)	**4.87** (2.18–9.47)	2.78 (1.09–6.03)
No. of contacts according to diagnosis			
Psychiatric symptoms/diagnoses	**3.26** (1.20–7.31)	**4.72** (1.71–9.84)	0.15 (0.00–1.16)
General symptoms/diagnoses	**1.29** (0.44–3.36)	**1.45** (0.54–3.54)	1.01 (0.34–1.16)
CVD symptoms/diagnoses	**1.13** (0.14–3.79)	**1.49** (0.23–4.28)	2.78 (0.68–7.45)
Other somatic symptoms/diagnoses	1.60 (0.49–4.03)	**2.31** (0.95–4.96)	2.05 (0.74–4.40)
Specialized somatic care usage per person‐year, median (25th‐75th percentiles)			
No. of contacts according to type			
Somatic admissions	**0.47** (0.19–0.89)	**0.64** (0.29–1.18)	0.52 (0.24–0.96)
Somatic emergency admissions	0.41 (0.17–0.79)	**0.52** (0.24–0.97)	0.45 (0.21–0.82)
Days in somatic hospital	**2.09** (0.85–4.80)	**3.02** (1.04–6.42)	2.57 (0.91–5.37)
Somatic out‐patient visits	**1.02** (0.28–2.21)	1.67 (0.71–3.27)	1.56 (0.60–3.19)
No. of contacts according to diagnosis			
Admissions with a CVD diagnosis	**0.25** (0.00–0.56)	0.32 (0.11–0.71)	0.35 (0.14–0.73)
Out‐patient visits with a CVD diagnosis	**0.00** (0.00–0.25)	**0.00** (0.00–0.42)	0.12 (0.00–0.49)

Abbreviations: CCI, Charlson comorbidity Index; CVD, cardiovascular disease; GP, general practitioner; SMI, severe mental illnesses; SD, standard deviation.

Bold figures: Significantly different from patients without SMI at a *P*‐value <0.05 when adjusting for sex and/or age group at death.

Data sources: The Norwegian Patient Registry (2008–2016), the Norwegian Directorate of Health system for the control and payment of health reimbursements in primary care (2008–2016), and the Norwegian Cause of Death Registry (2011–2016).

† ICD‐10 codes I60‐I69, G45‐G46 or H340, or ICPC‐2 codes K89‐K91.

Patients with SMI died at a mean age of 76 years, eight years younger than patients without SMI. No differences were noted in the main cardiovascular causes of death between the three diagnostic groups, with ischemic heart disease accounting for approximately 40% of all deaths in all groups. Patients with SMI had a lower likelihood of being diagnosed with arrhythmia, MI, valvular disease, and peripheral vascular disease, and patients with schizophrenia also had a lower likelihood of being diagnosed with hypertension. We found a similar prevalence of the diabetes diagnosis in patients with and without SMI, but a higher prevalence of diagnoses of substance abuse, obesity, and chronic obstructive pulmonary disease among patients with bipolar disorder.

Patients with schizophrenia had more GP telephone contacts but fewer GP visits, specialized somatic admissions, and somatic out‐patient visits than patients without SMI. Patients with bipolar disorder had a higher use of any form of GP contact and specialized somatic admissions than patients without SMI. Patients with SMI had a shorter time span from the first CVD examination in the observation period until cardiovascular death. Seventeen per cent of patients with schizophrenia, 11% of patients with bipolar disorder, and 8% of patients without SMI had their first CVD diagnosis recorded on the death certificate.

### Receipt of CVD‐related diagnostic tests

**Figure 1:**
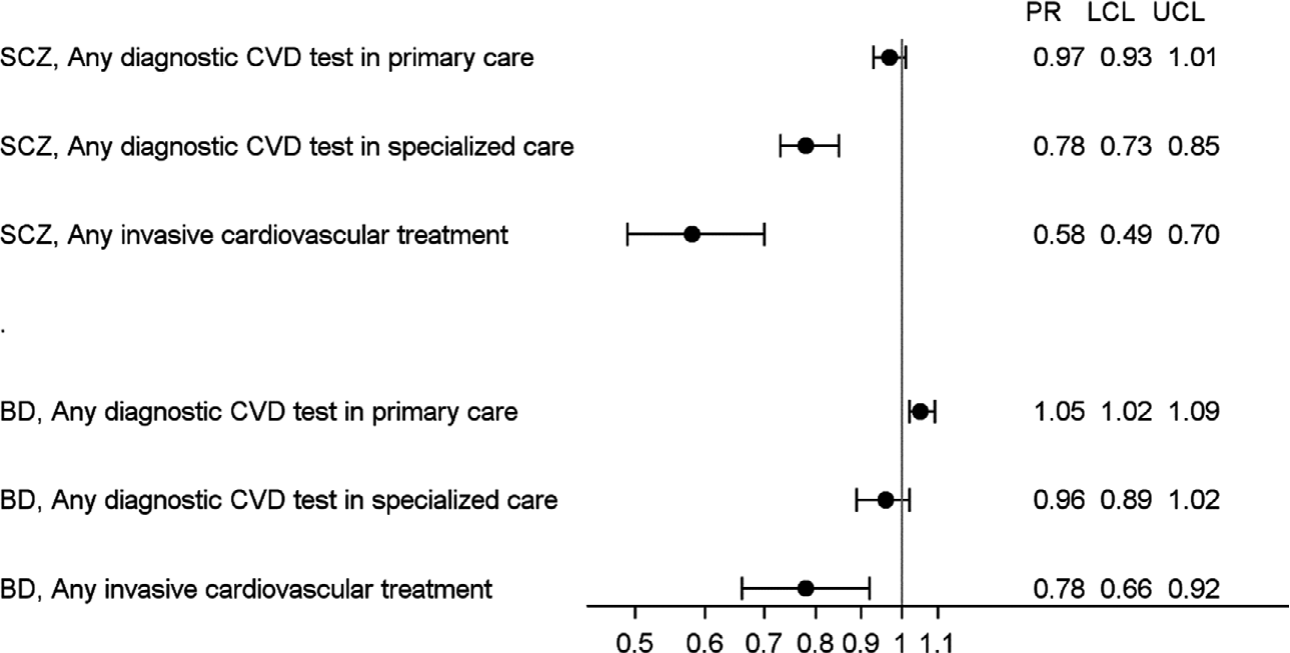
Prevalence ratios (PRs) adjusted for sex and age group with 95% lower (LCL) and upper (UCL) confidence limits for the receipt of diagnostic CVD tests or invasive cardiovascular treatment prior to cardiovascular death in individuals with schizophrenia (SCZ) or bipolar disorder (BD) compared to patients without severe mental illnesses after stratification according to the patient group and type of procedure. Data sources: The Norwegian Patient Registry (2008–2016), the Norwegian Directorate of Health system for the control and payment of health reimbursements in primary care settings (2008–2016), and the Norwegian Cause of Death Registry (2011–2016).

A diagnosis of SMI had no impact on the prevalence of receipt of any CVD‐related diagnostic test in primary care settings (Fig. 1). Compared to people without SMI, patients with bipolar disorder had a similar prevalence and patients with schizophrenia had a 22% lower prevalence of diagnostic cardiovascular examinations in specialized healthcare clinics.

According to the subgroup analyses, patients with schizophrenia had similar prevalence of ECG and diabetes testing in primary care settings, but a 61% lower prevalence of 24‐h blood pressure measurements, while patients with bipolar disorder had a 12–13% higher prevalence of blood glucose and HbA1c testing, but similar prevalence of ECG and 24‐h blood pressure measurements in primary care to patients without SMI (Table 3). A lower percentage of patients with schizophrenia underwent echocardiography (PR 0.77), coronary angiography (PR 0.66), or ultrasound of peripheral vessels (PR 0.44) tests compared to patients without SMI. Patients with bipolar disorder also exhibited reduced percentages of echocardiography (PR 0.90) and coronary angiography (PR 0.81), but a similar percentage of ultrasound of peripheral vessels compared to patients without SMI. Sex‐stratified analyses showed the same overall pattern.

**Table 3:** Adjusted prevalence ratios (PRs) with 95% confidence intervals (CI) for the receipt of diagnostic cardiovascular tests prior to cardiovascular death in individuals with schizophrenia or bipolar disorder compared to patients without severe mental illnesses after stratification by sex, patient group, and type of procedure

	All patients		Men			Women		
	PR†	95% CI	*n*	PR†	95% CI	*n*	PR†	95% CI
Diagnostic tests in primary care								
Electrocardiography (ECG)								
Schizophrenia	0.95	(0.89–1.02)	194	0.93	(0.84–1.03)	216	0.97	(0.88–1.06)
Bipolar disorder	1.06	(0.99–1.13)	173	1.05	(0.96–1.16)	212	1.06	(0.97–1.16)
24‐h blood pressure measurement								
Schizophrenia	**0.39**	(0.28–0.55)	14	**0.34**	(0.20–0.56)	17	**0.45**	(0.28–0.72)
Bipolar disorder	0.83	(0.65–1.07)	30	0.89	(0.63–1.26)	28	0.77	(0.54–1.10)
Glucose test								
Schizophrenia	1.00	(0.95–1.06)	228	1.00	(0.92–1.08)	264	1.00	(0.93–1.08)
Bipolar disorder	**1.13**	(1.07–1.19)	210	**1.17**	(1.09–1.26)	257	1.09	(1.02–1.17)
Glycated hemoglobin (HbA1c) test								
Schizophrenia	0.99	(0.91–1.07)	163	1.00	(0.89–1.12)	162	0.97	(0.86–1.10)
Bipolar disorder	**1.12**	(1.03–1.21)	149	**1.16**	(1.04–1.30)	162	1.07	(0.95–1.20)
Diagnostic tests in specialized health care								
Echocardiography								
Schizophrenia	**0.77**	(0.70–0.84)	138	**0.74**	(0.65–0.85)	139	**0.79**	(0.69–0.91)
Bipolar disorder	**0.90**	(0.82–0.98)	136	0.93	(0.82–1.05)	137	**0.84**	(0.74–0.96)
Coronary angiography								
Schizophrenia	**0.66**	(0.55–0.79)	58	**0.61**	(0.48–0.77)	39	**0.72**	(0.54–0.97)
Bipolar disorder	**0.81**	(0.67–0.96)	66	0.93	(0.75–1.15)	32	**0.64**	(0.46–0.89)
Ultrasound of peripheral vessels								
Schizophrenia	**0.44**	(0.32–0.61)	19	**0.46**	(0.30–0.72)	15	**0.41**	(0.25–0.67)
Bipolar disorder	1.09	(0.88–1.36)	35	1.04	(0.76–1.42)	39	1.12	(0.83–1.51)

Abbreviations: PR, prevalence ratio; CI, confidence interval; *n*, no. of patients who underwent the procedure.

Reference group: Individuals without schizophrenia or bipolar disorder who died from cardiovascular disease.

Bold figures: Significant association at a *P*‐value <0.05.

Data sources: The Norwegian Patient Registry (2008–2016), the Norwegian Directorate of Health system for the control and payment of health reimbursements in primary care (2008–2016), and the Norwegian Cause of Death Registry (2011–2016).

† Adjusted for sex, age at death, alcohol and drug use disorder, and somatic comorbidities.

### Receipt of invasive cardiovascular procedures

Patients with schizophrenia or bipolar disorder who died from CVD had 42% and 21% lower prevalence, respectively, of any invasive cardiovascular treatment (Fig. 1).

Subgroup analyses adjusted for the type of invasive treatment procedure showed a 35% lower prevalence of revascularization in patients with schizophrenia, but a similar prevalence of revascularization in patients with schizophrenia who were diagnosed with ischemic heart disease prior to death compared to patients without SMI (Table 4). We observed a 31% lower prevalence of any invasive arrhythmia treatment in patients with schizophrenia, but a similar prevalence in patients with schizophrenia who were diagnosed with arrhythmia prior to death compared to patients without SMI. Patients with bipolar disorder had a similar prevalence of revascularization and arrhythmia therapy to patients without SMI, regardless of a diagnosis of ischemic heart disease or arrhythmia. Patients with schizophrenia or bipolar disorder had 57% and 63% lower prevalence, respectively, of vascular surgery than patients without SMI. Patients with bipolar disorder who were diagnosed with peripheral vascular disease or carotid artery stenosis prior to death had a 50% lower prevalence of vascular surgery than patients without SMI. Sensitivity analyses essentially provided the same estimates for PR as the primary analyses (Figure S1).

**Table 4:** Adjusted prevalence ratios (PRs) with 95% confidence intervals (CI) for the receipt of invasive cardiovascular treatment prior to cardiovascular death in individuals with schizophrenia or bipolar disorder compared to patients without severe mental illnesses after stratification according to the patient group, inclusion criteria, and type of procedure.

	Patients	Patients who underwent the procedure	PR†	95% CI
Percutaneous coronary intervention (PCI) or coronary artery bypass surgery (CABG)				
Patients with CVD as the underlying cause of death				
Schizophrenia	814	54	0.65	(0.50–0.84)
Bipolar disorder	673	54	0.79	(0.61–1.01)
Patients diagnosed with IHD prior to death				
Schizophrenia	327	54	0.82	(0.65–1.03)
Bipolar disorder	272	54	0.94	(0.75–1.18)
Arrhythmia therapy				
Patients with CVD as the underlying cause of death				
Schizophrenia	814	42	0.69	(0.51–0.92)
Bipolar disorder	673	44	0.87	(0.65–1.15)
Patients diagnosed with arrhythmia prior to death				
Schizophrenia	298	42	0.86	(0.65–1.14)
Bipolar disorder	262	44	0.97	(0.74–1.27)
Peripheral vascular surgery				
Patients with CVD as the underlying cause of death				
Schizophrenia	814	19	0.43	(0.28–0.68)
Bipolar disorder	673	14	0.37	(0.22–0.62)
Patients diagnosed with peripheral vascular disease prior to death				
Schizophrenia	101	19	0.73	(0.49–1.09)
Bipolar disorder	103	14	0.50	(0.31–0.82)

Abbreviations: PR, prevalence ratio; CI, confidence interval; CVD, cardiovascular disease; IHD, ischemic heart disease.

Reference group: Individuals without schizophrenia or bipolar disorder who died from cardiovascular disease.

Bold figures: Significant association at a *P*‐value <0.05.

Data sources: The Norwegian Patient Registry (2008–2016), the Norwegian Directorate of Health system for the control and payment of health reimbursements in primary care (2008–2016), and the Norwegian Cause of Death Registry (2011–2016).

† Adjusted for sex, age at death, alcohol and drug use disorder, and somatic comorbidities.

## Discussion

In this nationwide registry‐based study of all cardiovascular deaths among adult Norwegian residents from 2011 to 2016, we observed a comparable use of CVD‐related diagnostic tests in primary care settings, but a lower use of diagnostic cardiovascular examinations in specialized somatic healthcare settings among patients with schizophrenia, and a lower use of invasive cardiovascular treatments among patients with schizophrenia or bipolar disorder compared to patients without SMI. However, patients with schizophrenia or bipolar disorder who had a recognized and diagnosed CVD had similar prevalence for invasive cardiovascular therapy as patients without SMI.

Our findings show similarities with other studies on related topics. Patients with schizophrenia have a similar likelihood of ECG and higher likelihood of HbA1c testing compared to controls with a similar somatic comorbidity 25. The findings for 24‐h blood pressure measurements in primary care settings are consistent with previous studies of people with schizophrenia 8, 13, 32 or bipolar disorder 22, 24, 33. A similar result was reported for diabetes testing in primary care settings 23, 24, 34, 35, indicating that GPs are well aware of the increased cardiometabolic risk in individuals with SMI.

A decreased prevalence of coronary angiography in patients with schizophrenia or bipolar disorder is consistent with earlier studies 7, 15, 16, 36, 37, 38, 39. Little is known about the extent to which these patients receive echocardiography and ultrasound of peripheral vessels, but patients with SMI are less likely to undergo a left ventricular ejection fraction assessment, for which echocardiography is often used 40.

The lower prevalence of invasive cardiovascular procedures in patients with schizophrenia or bipolar disorder is consistent with previous studies 39, 41. Overall, a lower (schizophrenia) or similar (bipolar disorder) prevalence of revascularization was observed in patients with SMI, but similar prevalence of revascularization was observed in patients with and without SMI with acknowledged ischemic heart disease. The latter finding is consistent with a nationwide Swedish study 17, and a recent Danish study that did not observe a difference in post‐MI treatment in patients with and without schizophrenia who have undergone coronary angiography 42, as well as studies showing a similar likelihood of revascularization in patients with ischemic heart disease and bipolar 43 or mood disorders 20. The finding of a similar prevalence of revascularization in patients with SMI diagnosed with ischemic heart disease is also compatible with a study of the general Norwegian population, showing that lower revascularization rates among patients with a low education level are explained by differences in the receipt of coronary angiography 44.

With the exception of revascularization, the receipt of invasive cardiovascular treatment by individuals with SMI has been rarely described in the literature 45. However, an Israeli study reported a 50% reduced likelihood of cardiac pacemaker implantation in patients with schizophrenia 39 and a US study described a reduced likelihood of receiving major surgery, including vascular surgery, among individuals with SMI, particularly patients with schizophrenia 41. These findings are consistent with the data from our study and support the hypothesis that patients with SMI receive fewer cardiovascular procedures in specialist healthcare settings than people without SMI.

Disease‐related factors, such as a limited and disorganized self‐care capacity, poor communication skills, self‐stigma, depression, and social isolation, particularly in patients with schizophrenia, may lead to delayed presentation or diagnosis, a failure to attend visits and missed referrals. According to previous studies, older patients with SMI may be particularly less likely to seek health care for CVD 46. Increased pain tolerance 47 and an increased risk of silent CVD 48 have been reported in individuals with schizophrenia, which might prevent them from seeking health care in a timely manner. Because of the increase in early cardiovascular mortality 49, 50, individuals with SMI may die at ages when healthcare providers do not usually suspect CVD. Prevailing risk prediction algorithms for CVD have been shown to underestimate the risk in individuals with schizophrenia 51. Furthermore, shared genetic risk factors for both schizophrenia and CVD 52 may imply a more malignant course of CVD, with less time to recognize symptoms, in patients with SMI. Users of antipsychotic medication are at higher risks of adverse cardiac effects such as arrhythmias, deviations in blood pressure, heart failure, myocarditis, and cardiomyopathy, which might lead to sudden cardiac death 53.

Provider‐level explanations include time constraints, complexity in the organization of health care, and misattribution of physical symptoms to the mental illness 54. Discomfort with or stigmatizing attitudes toward patients with SMI among healthcare providers have also been reported 55, particularly regarding schizophrenia 56, and these attitudes may affect the propensity of diagnostic testing and treatment. The lower prevalence of cardiovascular examinations was particularly pronounced for procedures that are time‐consuming or require physical contact with the patient (such as 24‐h blood pressure measurements, echocardiograms, coronary angiography, and ultrasound of peripheral vessels) in the present study. This decrease in the prevalence of these procedures might be due to patient‐related factors and stigmatizing attitudes among providers.

Despite their younger age, people with SMI have more comorbidities 57 and higher risks of postoperative complications 58 and mortality 17 following cardiac events. Disparities in invasive cardiovascular treatment may therefore also reflect clinicians’ uncertainty about treatment risks or compliance with postoperative care. Lack of consent or a lack of capacity to provide informed consent may also be an issue, as noted in a recent study investigating decisions about cardiac examinations and treatment among patients with schizophrenia 42.

Finally, a mismatch between patients' healthcare needs and the fragmented organization of health care may be an important barrier to somatic health care 59. The current healthcare system relies on the individual’s ability to initiate contact with healthcare providers. The ability to navigate between psychiatric, primary, and specialized somatic healthcare systems may be a challenge, particularly for patients with SMI, and is potentially aggravated by a lack of clarity as to which health provider is responsible for monitoring the physical health of this patient group 60. Both patients and mental health staff suggested the need for a less fragmented healthcare system when asked about potential improvements to the prevention and treatment of physical diseases among individuals with SMI 59, 61.

Paradoxically, people with bipolar disorder who died from CVD died at the same age as patients with schizophrenia and at a much younger age than subjects without SMI, despite having a higher overall healthcare usage and generally a higher education level and lower level of functional impairment than people with schizophrenia 1. We can only speculate on the causes for this finding. Comorbid alcohol use disorder, which is more prevalent in individuals with bipolar disorder and is associated with a lower prevalence of specialized somatic examination and treatment, may be part of the explanation. A higher rate of somatic comorbidities in patients with bipolar disorder, including conditions that complicate invasive cardiovascular treatment, may be another explanation 62.

### Strengths and limitations

The strengths of this study include the use of a nationwide sample with complete diagnostic data from both primary and specialized healthcare settings, the inclusion of patients with an assumed similar severity of CVD, available data on somatic comorbidities and substance use disorders, and the use of extensive sensitivity analyses to strengthen our conclusions. However, some limitations should be mentioned.

First, we did not have access to data on physicians' assessments of risk factors or data on referrals or patients' possible lack of consent to undergoing clinical examinations and treatment. We were thus unable to precisely determine where and why inequalities occur along the clinical pathway. We also lacked information on pharmacological approaches used to prevent and treat CVD, radiological procedures (such as computed tomographic angiography and ultrasound of peripheral vessels), and prehospital procedures during transportation to the hospital (such as ECG or thrombolysis). We did not know the specific indication for blood tests performed as part of broad batteries covering several biomarkers. Thus, we may have underestimated the level of screening/monitoring of CVD risk factors in all patients, but the underestimation was probably greater in patients without SMI, as patients with SMI are monitored for adverse effects of psychopharmacological medications. Additionally, very common procedures, such as ordinary blood pressure measurements or ECG, may be underreported in administrative data, but this underreporting would not likely differ systematically between patients with and without SMI.

Second, we lacked information on the severity of CVD (such as ST‐segment elevation MI and extent of coronary disease). An equal severity of CVD might be approximated by the observation that all patients died from CVD. Previous studies have reported a similar severity of MI among patients with schizophrenia 17, and a similar 17 or higher 43 severity of MI among patients with bipolar disorder, which implies a possible underestimation of the differences in access to invasive cardiovascular treatments for patients with bipolar disorder. We also lacked information on the time from symptom onset and travel time to hospital, which may have differentially affected treatment options in patients with and without SMI.

Third, we lacked information on the socioeconomic status of the patients before their prodromal phase (e.g., their parents' educational attainment). Our results might therefore be confounded by social stratification in terms of treatment usage.

Fourth, the validity of registry diagnoses is unknown for some disease categories. Previous studies have reported a high level of agreement between clinical and research‐based diagnoses of SMI 63 and valid information regarding stroke 64 and intracranial hemorrhage 65 in the Patient Registry. Studies from other Nordic countries have observed a high level of diagnostic accuracy of cardiovascular diagnoses in general 66, 67, atrial fibrillation and flutter 68, and intracerebral hemorrhage 69, but questionable validity of heart failure 70 and peripheral arterial disease diagnoses 71. Diagnoses in the reimbursement database have not been validated, but diagnoses have been found to be valid in general practice registries in other countries, particularly for chronic diseases 72. The registered cause of death was not validated by autopsy for the large majority of patients in our study. However, the Cause of Death Registry has been shown to provide high‐quality information on causes of death 73. A Norwegian study from 2011 reported very good agreement between mortality statistics and autopsy findings for both coronary heart disease and stroke in the registry 74. The most common error in mortality statistics is that immediate or intermediate causes of death are registered as the underlying cause of death 75. However, we obtained similar results when we included all deaths with CVD as the underlying or contributing cause of death in the sensitivity analysis. We lacked data on sudden death (ICD‐10 code R98), which may imply unreported cardiovascular deaths. However, only 2% of underlying causes of death in Norway from 2011 to 2016 were assigned to unknown or unspecified causes (R96–R99) 76. Studies have reported a longer postmortem time before discovery in individuals with schizophrenia compared to patients with other disorders 77, which possibly affects the reliability of cause of death codes in these individuals.

Fifth, a longer time series would have been preferable, but was not possible with the available registries in Norway.

Finally, external validity may be limited to individuals with severe CVD in countries with publicly funded and readily available health services for both somatic and mental disorders.

### Clinical implications

In this nationwide study, we observed a lower prevalence of specialized cardiovascular examinations prior to cardiovascular death in patients with schizophrenia and patients with bipolar disorder, despite high levels of primary and somatic healthcare use. Vulnerable individuals may therefore be deprived of medically indicated cardiovascular treatments. In patients diagnosed with CVD, however, we observed a similar prevalence of invasive cardiovascular treatment among patients with and without SMI.

Based on these results, the underdiagnosis and underuse of specialized cardiovascular examinations, rather than poor access to primary care or restraints because of contraindications or perceived non‐compliance with postoperative care, are the main obstacles to more equal access to cardiovascular health care for patients with SMI. The window of opportunity for improvement appears to be the interface between GPs and somatic hospital departments. The healthcare disparities identified in our study call for a proactive, flexible, and tailored approach for the timely and specific diagnosis and treatment of CVD in patients with SMI. Possible remedies for these disparities may be economic incentives for performing cardiovascular examinations on patients with SMI in primary and specialized healthcare settings, and incentives for specialized cooperation arenas between psychiatric and somatic caregivers. Specific, standardized algorithms for diagnosis and treatment should also be implemented, because cardiovascular disease may have a more malignant disease course in these patients, with less time from the first occurrence of symptoms to development of severe sequelae or death.

## Declaration of Interest

The authors have not conflicts of interest to declare.

## Supporting information


**Figure S1** **.** Results of sensitivity analyses.
**Table S1** **.** Definitions of patient groups.

## Data Availability

The data that support the findings of this study are available from the Norwegian Patient Registry, the Norwegian Directorate of Health (the KUHR database), and the Norwegian Cause of Death Registry. Restrictions apply to the availability of these data, which were used under license for this study. For information on how to access the data, see https://www.helsedirektoratet.no/tema/statistikk-registre-og-rapporter/helsedata-og-helseregistre/norsk-pasientregister-npr/sok-om-data-fra-npr.
