# Supplementary material for: Diagnostic tests and treatment procedures performed prior to cardiovascular death in individuals with severe mental illness
Source: Acta Psychiatr Scand. 2020 Feb 29;141(5):439–51. doi: 10.1111/acps.13157 (PMC7317477; doi:10.1111/acps.13157)
Supplement: Supplementary file 1 — Figure S1 . Results of sensitivity analyses. Table S1 . Definitions of patient groups. [file ACPS-141-439-s001.docx]

Table S1 Definitions of patient groups

| **Patient group** | **ICD-10 codes** | **ICPC-2 codes** |
| --- | --- | --- |
| Congestive Heart Failure | I43, I50, I09.9, I11.0, I13.0, I13.2, I25.5, I42.0, I42.5-I42.9, P29.0 | K71, K77 |
| Cardiac arrhythmias | I47-I49, I44.1-I44.3, I45.6, I45.9, R00.0-R00.1, R00.8, T82.1, Z45.0, Z95.0 | K05, K78-K80 |
| Valvular disease | I05-I08, I34-I39, A52.0, I09.1, I09.8, Q23.0-Q23.3, Z95.2-Z95.4 | K83 |
| Pulmonary circulation disorders | I26-I27, I28.0, I28.8-I28.9 | K82, K93 |
| Peripheral vascular disorders | I70-I71, I73.1, I73.8-I73.9, I77.1, I79.0, I79.2, K55.1, K55.8-K55.9, Z95.8, Z95.9 | K92 |
| Hypertension, uncomplicated | I10 | K86 |
| Hypertension, complicated | I11-I13, I15 | K87 |
| Paralysis | G81-G82, G04.1, G11.4, G80.1-G80.2, G83.0-G83.4, G83.9 | - |
| Other neurological disorders | G10-G13, G20-G22, G32, G35-G37, G40-G41, R56, G25.4-G25.5, G31.2, G31.8-G31.9, G93.1, G93.4, R47.0 | N07, N86-N88 |
| Chronic pulmonary disease | J40-J47, J60-J67, I27.8-I27.9, J68.4, J70.1, J70.3 | K82, R79, R95-R96 |
| Diabetes, uncomplicated | E10.0-E10.1, E10.9-E11.1, E11.9-E12.1, E12.9-E13.1, E13.9-E14.1, E14.9 | T90 |
| Diabetes, complicated | E10.2-E10.8, E11.2-E11.8, E12.2-E12.8, E13.2-E13.8, E14.2-E14.8 | T89 |
| Hypothyroidism | E00-E03, E890 | T86 |
| Renal failure | N18-N19, I12.0, I13.1, N25.0, Z49.0-Z49.2, Z94.0, Z99.2 | - |
| Liver disease | B18, I85, K70, K72-K74, I86.4, I98.2, K71.1, K71.3-K71.5, K71.7, K76.0, K76.2-K76.9, Z94.4 | D97 |
| Peptic ulcer disease excluding bleeding | K25.7, K25.9, K26.7, K26.9, K27.7, K27.9, K28.7, K28.9 | D85-D86 |
| AIDS/HIV | B20-B22, B24 | B90 |
| Lymphoma | C81-C85, C88, C96, C90.0, C90.2 | B72, B74 |
| Metastatic cancer | C77-C80 | - |
| Solid tumor without metastasis | C0-C1, C6, C20-C26, C30-C34, C37-C41, C43, C45-C58, C70-C76, C97 | D74-D75, D77, L71, N74, R84-R85, T71, U75-U77, W72, X75-X77, Y77-Y78 |
| Rheumatoid arthritis/collagen vascular diseases | M05-M06, M08, M30, M32-M35, M45, L94.0-L94.1, L94.3, M12.0, M12.3, M31.0-M31.3, M46.1, M46.8-M46.9 | L88 |
| Coagulopathy | D65-D68, D69.1, D69.3-D69.6 | - |
| Obesity | E66 | T82-T83 |
| Weight loss | E40-E46, R64, R63.4 | T08 |
| Fluid and electrolyte disorders | E86-E87, E22.2 | T11 |
| Blood loss anemia | D50.0 | - |
| Deficiency anemia | D51-D53, D50.8-D50.9 | B80-B81 |
| Alcohol abuse | F10, E52, T51, G62.1, I42.6, K29.2, K70.0, K70.3, K70.9, Z50.2, Z71.4, Z72.1 | P15, P16 |
| Drug abuse | F11-F16, F18-F19, Z71.5, Z72.2 | P18-P19 |
| Lipidemia | E78 | T93 |
| Dementia | F00-F03, G30, F05.1, G31.1 | P70 |


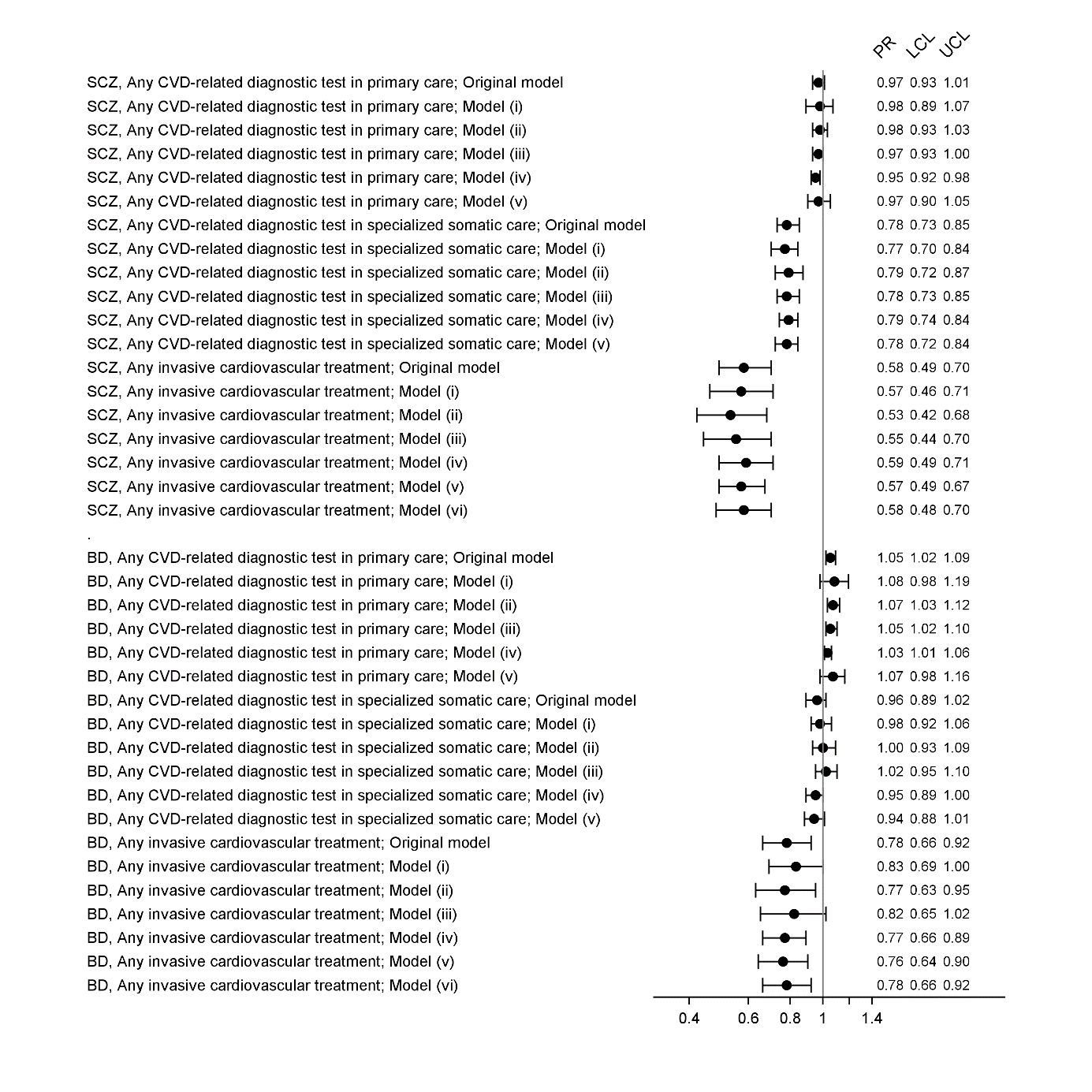


Figure S1 Results of sensitivity analyses. Adjusted Prevalence Ratios (PR) with 95% lower (LCL) and upper (UCL) Confidence Limits for receipt of diagnostic CVD tests or invasive cardiovascular treatment prior to cardiovascular death in individuals with schizophrenia (SCZ) or bipolar disorder (BD), according to patient group, health care sector and type of procedure.

Data sources: The Norwegian Patient Registry (2008-2016), the Norwegian Directorate of Health’s system for control and payment of health reimbursements in primary care (2008-2016) and the Norwegian Cause of Death Registry (2011-2016).

Model (i): Excluding patients diagnosed with dementia

Model (ii): Excluding patients aged 80 and above

Model (iii): Excluding patients with the ambiguous affective disorder diagnosis (ICPC-2 code P75) from the BD group

Model (iv): Including also cases with CVD as contributing cause of death

Model (v): Adjusting for person-years of observation

Model (vi): Excluding persons who died at their first CVD encounter in the period
